# Segmentation of Endothelial Cell Boundaries of Rabbit Aortic Images Using a Machine Learning Approach

**DOI:** 10.1155/2011/270247

**Published:** 2011-06-28

**Authors:** Saadia Iftikhar, Andrew R. Bond, Asim I. Wagan, Peter D. Weinberg, Anil A. Bharath

**Affiliations:** ^1^Department of Bioengineering, Imperial College London, London SW7 2AZ, UK; ^2^Bristol Heart Institute, Bristol Royal Infirmary, Bristol BS2 8HW, UK; ^3^Laboratoire d'InfoRmatique en Image et Systèmes d'information, Institut National des Sciences Appliquées de Lyon, LIRIS INSA De Lyon, 69621 Villeurbanne, France

## Abstract

This paper presents an automatic detection method for thin boundaries of silver-stained endothelial cells (ECs) imaged using light microscopy of endothelium mono-layers from rabbit aortas. To achieve this, a segmentation technique was developed, which relies on a rich feature space to describe the spatial neighbourhood of each pixel and employs a Support Vector Machine (SVM) as a classifier. This segmentation approach is compared, using hand-labelled data, to a number of standard segmentation/thresholding methods commonly applied in microscopy. The importance of different features is also assessed using the method of minimum Redundancy, Maximum Relevance (mRMR), and the effect of different SVM kernels is also considered. The results show that the approach suggested in this paper attains much greater accuracy than standard techniques; in our comparisons with manually labelled data, our proposed technique is able to identify boundary pixels to an accuracy of 93%. More significantly, out of a set of 56 regions of image data, 43 regions were binarised to a useful level of accuracy. The results obtained from the image segmentation technique developed here may be used for the study of shape and alignment of ECs, and hence patterns of blood flow, around arterial branches.

## 1. Introduction

Atherosclerosis is the major cause of cardiovascular morbidity and mortality, with underlying pathological processes that may begin during childhood [1, 2]. Although the exact causes of atherosclerosis are not clear, it is thought to involve lipoprotein influx into the wall, across the endothelium, and chronic inflammation. Over time, lipids accumulate in the inner wall and plaques develop, resulting in reduction or blockage of blood flow. Eventually, this condition can lead to heart attacks and strokes [3, 4]. A striking feature of the disease is its nonuniform distribution within the arterial system. This is most evident in regions of branching and curvature and has therefore been attributed to spatial variation in mechanical forces, particularly the haemodynamic wall shear stress exerted on the endothelium by the flow of blood. Near-wall blood velocity, on which shear stress depends, cannot be accurately measured by direct techniques. However, ECs form a monolayer between the blood and arterial wall [3] that is regulated by haemodynamic forces through flow-mediated signal transduction [3, 5]. Of relevance to the present study, endothelial cells and their nuclei align with the predominant flow direction and elongate in response to increased shear. Therefore, ECs can be viewed as “flow sensors,” and their shape has been used to assess patterns of wall shear stress in previous studies, [6, 25], including our studies aimed at understanding why the pattern of disease around aortic branches changes with age [8, 9].

In the present study, we developed methods for automated analysis of ECs morphology [9]. The first step is to detect the boundaries of the cells against the background of stained images. This is difficult in cases where the noise level is high and the image contrast is poor. This has motivated us to use “Support Vector Machines” (SVMs) as a classifier because recent work has shown this approach to outperform many conventional classifiers [10]. In this paper, we describe the visual features and the subsequent application of the SVM as a classifier to detect thin boundaries of endothelial cells.

## 2. Data Acquisition

**Figure 1:**
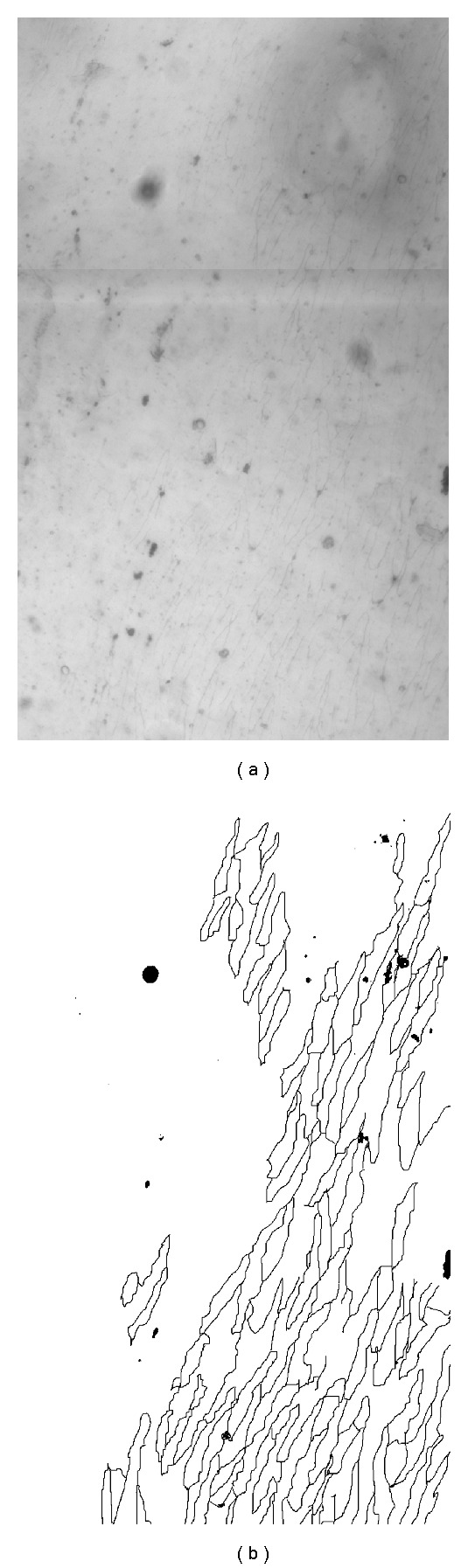
Original sample of ECs and corresponding manually traced image using the GNU Image Manipulation Program (GIMP) http://www.gimp.org/.

Endothelial monolayers were stripped from the descending thoracic aortas of rabbits by a modification of the Häutchen procedure of Bond et al. [9] and Hirsch et al.[11]. This involves pressing the endothelial surface of the opened aorta against double-sided adhesive tape adhered to the surface of a microscope slide. The aorta is then pulled away, leaving the endothelium attached to the slide. Aortas were obtained from three male New Zealand White rabbits (Harlan Interfauna strain), one mature and two immature, that had been perfused in situ with 10% neutral buffered formalin at physiological pressure for 90 s followed by 20 mL of silver nitrate solution (2.5 mg/mL, Sigma), followed by further formalin fixation for 30 mins. All animal procedures complied with the Animals (Scientific Procedures, UK) Act 1986. The silver nitrate was used to stain the boundaries between neighbouring cells. Cell boundaries were examined around the origins of seven intercostal arteries from the descending thoracic aortas of the three rabbits. A montage of images of the area around each branch mouth was obtained using a Zeiss Axioplan microscope [9]. The spatially varying sensitivity of the microscope and camera system resulted in shading of the individual images; in Section 3, we describe the correction of these distortions, though correction is not needed in our final system. Each montage was then divided into subregions; the subregions each corresponded to an arterial area of approximately 660 × 1100 pixels, and they were located in a 3 × 3 grid centred on the branch mouth. The central element of the 9-element grid was not used, since it was largely occupied by the branch mouth, giving eight regions at each of the seven branches, and hence 56 regions in total. The images corresponding to these 56 regions comprised the data set to which the current analysis was applied. One of the sample images (with a size of 660 × 1100 pixels) taken from one of the montages is shown in Figure 1(a) and its manually traced counterpart in Figure 1(b).

## 3. Segmentation of Endothelial Cell Boundaries

The cell boundary labelling or segmentation of ECs can be described as a binary classification problem in the sense that cell boundary pixels should be labelled as object or foreground and non-boundary pixels should be labelled as background [13]. Each of the 56 datasets contained more than 500 cells, giving >25,000 cells requiring analysis. It is extremely tedious to trace the boundaries manually for tens of thousands of cells, and so a number of different methods was applied to attempt the segmentation of boundary pixels automatically.

In order to assess the applicability of intensity thresholding to the task, we analysed the intensity distributions of image data after applying intensity correction based on background subtraction using a least-squares polynomial fit to a second-order spatial illumination model. For images that fell across boundaries of a montage, we also used the average values of intensity on either side of the boundary to perform additive compensation of intensity differences in different acquisitions. Then, by using hand labelling of image and background data, we estimated the parameters of a standard mixture model:

(1) $$\begin{array}{c} \begin{array}{cc} P\left(i\right) & =P_{O}P\left(i\;|\;O\right)+P_{b}P\;\left(i\;|\;b\right), \end{array} \end{array}$$

where *P*_*O*_ and *P*_*b*_ are the prior probabilities of the pixels and *P*(*i* | *O*) and *P*(*i* | *b*) are conditional probabilities of boundary and background, respectively. *P*(*i*) is the weighted sum of *P*(*i* | *O*) and *P*(*i* | *b*) [12] and “explains” the histograms of all pixel intensities pooled together.

**Figure 2:**
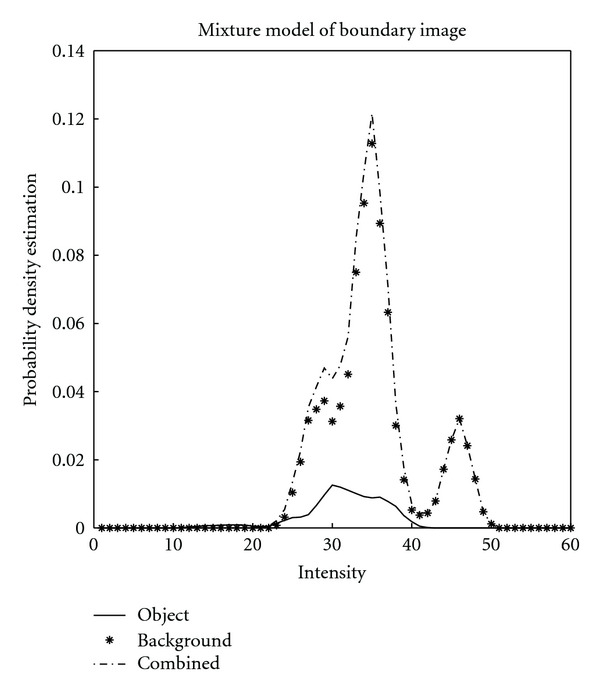
Estimated probability density functions of intensities of cell boundary pixels (solid line) and those that lie off the cell boundaries (background, asterisks), using manually labelled, intensity corrected data. The graph illustrates that the pixel intensity distributions of boundary and non-boundary pixels overlap strongly, even over small spatial scales. Adapted from [12].

As shown in Figure 2, the conditional probability density functions of background and cell boundary pixels overlap with each other strongly. It is thus hard to set a global threshold which distinguishes between background and boundary pixels; we have previously described results of our initial work on the same data using adaptive thresholding following quadratic-based trend removal, a type of adaptive high-pass filtering [12].

We therefore developed a technique with the help of a class of machine learning algorithms known as Support Vector Machines (SVMs) to computationally detect the boundaries of the images. The method is attractive in that it is able to handle the weak boundary signals in our data, and does not require explicit edge-linking or explicit illumination correction—these latter two modules of classical segmentation are implicit properties in the choice of features. Once such labellings are obtained, it is fairly straightforward to apply a variety of shape analysis techniques on the boundary representations to study the morphology of cells under the effects of biological flow [9]. The rest of the paper describes the method we have developed for automatically detecting the cell boundaries using SVMs and some evaluations of its performance.

## 4. A Segmentation Algorithm for Cell Boundaries

**Figure 3:**
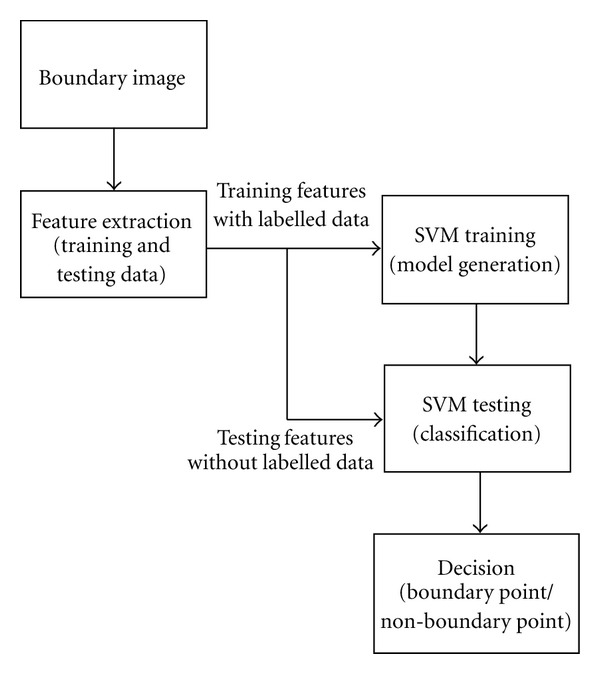
Flow chart of segmentation algorithm based on SVM for endothelial cell images.

The flow chart of our algorithm is given in Figure 3. Features are extracted from the cell boundary image and mapped into feature space. To train the classifier, features are extracted from a training image for which there exists manually labelled data. Then, using the input feature space generated from the training image and an SVM kernel, a decision model is generated; this model is then applied to segment other boundary images. The SVM classifies image pixels into two different classes: boundary and non-boundary. It is useful to consider the complexity of this problem, and why the selection of patch-based features, though increasing feature space dimensions, helps solve the segmentation problem.

**Figure 4:**
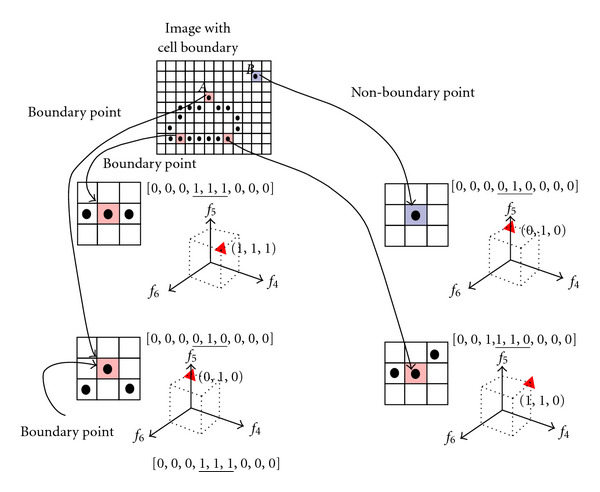
Mapping of neighbourhoods of points into the components of a 9-element feature vector, [*f*_1_, *f*_2_,…, *f*_9_]. Four candidate points are considered, three corresponding to true boundary points, and one corresponding to an isolated noise pixel (*B*) that has an intensity similar to those of the boundary points. Note that if we plot where the points in a three-dimensional subspace of 9-dimensional space lie (red triangles), we find that pixels *A* and *B* fall in the same place in the three dimensional subspace formed by components *f*_4_, *f*_5_, and *f*_6_; this means that this subspace cannot be sufficient to classify pixels correctly. Creating a feature vector containing all 8 neighbouring pixels' intensities, and that of the candidate pixel, provides a much better chance of successfully teaching an SVM to recognise boundary pixels from non-boundary pixels, but even this is not sufficient for low signal-to-noise conditions (see text for details).

Figure 4 illustrates the complexity of learning to detect boundary pixels. Since we cannot rely purely on the image intensity of a single pixel in order to classify it—the basis of thresholding approaches—the decision-making process on the correct classification for each pixel needs to consider the intensity of its neighbours as well. Figure 4 shows four different configurations of pixels, similar to the “cliques” of Markov-Random Fields (MRFs), and considers a subspace of the full 9-dimensional space of the 3 × 3 patch represented by the intensities along a horizontal line of three pixels centred on the pixel to be classified. Three of these pixels are valid boundary points, and the fourth (*B*) is an isolated pixel that has a boundary-like intensity, but contains no neighbouring pixels to “support” it. The consideration of three pixels is not enough to allow a correct decision to be made concerning pixel *A* (boundary) and pixel *B* (non-boundary), as they lie in the same place in 3-dimensional feature space. Including all the eight nearest neighbours of a pixel to be classified provides a partial solution, but the separation of classes in a 9-dimensional space is more difficult, and almost impossible using a linear classifier such as a Fisher criterion [14]. This is the motivation for using SVMs. Through the use of kernels that generate nonlinear combinations of variables, SVMs are able to find decision boundaries for the classes that are not easily separable in the original space.

Other approaches to this problem might include algorithms to perform boundary linking, based on directions of gradients. Such approaches might work for images in which the object boundaries correspond to edges of regions which contain different average pixel intensities on either side of the boundary; for the case of thin structures, second-order directional derivatives or phase-invariant measures of orientation are more appropriate for estimating structure direction to perform edge linking. Even so, creating an edge-linking algorithm to correctly handle the very complex spatial patterns formed by junctions between cells is no trivial task, and it is better to pursue a training approach which can learn how to appropriately classify pixels based on spatial configurations of neighbours.

However, given the nature of the poor signal-to-noise levels in the images, even a 3 × 3 neighbourhood of pixel values is not sufficient as a feature vector; and whilst a 5 × 5 neighbourhood might provide more information that would allow, say, pixels lying on gaps in boundaries to be correctly classified, it would require an increase in the dimensions of the feature vector, more training and testing data, and also would incur higher computational cost in training and classification. Instead, we resort to generating features that group information from a wider area, for example, through spatial filtering, to compactly capture information over a larger neighbourhood which explicitly describes local orientations. We have taken an intuitive approach to selecting features, but have validated it using a feature selection approach.

### 4.1. Selection of Visual Features for the SVM

We have explored a rich set of possible feature vector combinations, using up to 75 components in the feature vector, but do not reproduce them all here. Rather, we take the strongest feature vector components to illustrate the process of feature selection using the principle of minimum Redundancy, Maximum Relevance (mRMR) [15]. This is illustrative, as it shows that some features are much more informative than others. Although we have used feature selection to assess some possible features, one has to be careful in assessing features individually. As illustrated by Figure 4, some features provide strong discriminating power only when included as a collection over space—a patch-based approach, whereby some components of the feature vector are taken from a property that is estimated at different locations over neighbourhood space. This is the case for the patch-based information such as the intensity and orientation dominance field. Whilst linear classifiers cannot learn the tortuous decision boundaries suggested by the training data in, say, 9-dimensional space that are required to satisfy orientation invariance, the SVM is able to find separating hyperplanes which generalise well [16] from relatively few examples.

### 4.2. Feature Generation

**Figure 5:**
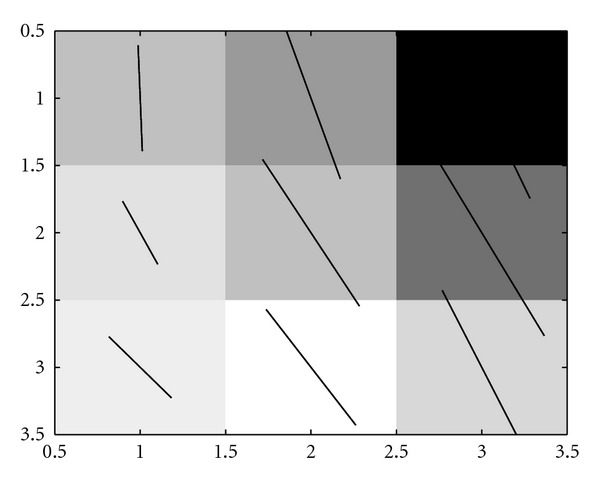
A sample of a 3 × 3 neighbourhood of pixels with the orientation field of candidate pixels overlaid with black lines.

Each pixel in the image is mapped to a 33-dimensional feature vector by extracting information from a 3 × 3 neighborhood of the central pixel:

- nine intensity elements extracted from 3 × 3 nearest neighbors;
- eighteen features extracted from 3 × 3 nearest neighbors of an orientation dominance field [17]; the magnitude of the vectors in this field ranges from 0 to 1. The magnitude of the values can be used as an indication of anisotropy, in which strongly isotropic neighbors will produce values near to 0 and strongly anisotropic neighbors will produce values near to 1; an example of this field over a 3 × 3 patch is shown in Figure 5. These features are quasi-invariant to slow background illumination changes because they are generated from band pass filters;
- six statistical features generated from the 3 × 3 intensity neighborhood of a candidate pixel, given in Table 1.

The 33-dimensional feature vector given in (2) is then described for each pixel in the image in both the training and testing phases of the SVM

(2) $$\begin{array}{cc} \vec{FV_{n}} & =\left[I_{\left(3\times 3\right)},{\vec{O}}_{\left(3\times 3\right)},\text{Med, Range},E,M_{2,3,4}\right]. \end{array}$$

The feature vector components are normalised by removing the mean of each feature and dividing the result by the standard deviation of each feature. No length normalisation is performed outside of the SVM.

### 4.3. Feature Relevance

We used the minimum Redundancy, Maximum Relevance (mRMR) measure to evaluate the importance of, and thereby select, the components of the feature vector input to the SVM. As implied by Figure 4, some features should not be separated or assessed on their own. The result of mRMR is not repeated here, because it is performed against a very large number of neighbourhood features, including edge filters, statistical moments of intensities, and order-statistic neighbourhood measures. As a general rule, we found that orientation dominance, pixel intensities, and statistical moments were the most discriminating, (Table 2), and so we included these in the feature vector we have used for segmentation.

A certain degree of caution is required in interpreting mRMR results: individual neighbourhood pixels may be split apart in ranking of mRMR methods. Thus, for example, if pixels from an 3 × 3 patch are all included in the mRMR, they may be separated in the ranked list of relevance obtained from the standard mRMR algorithm. When such features, obtained from different pixel locations in a feature space image around the candidate pixel, are included in an mRMR analysis, one should note the frequency with which the different locations of one property occur in, say, the top *K* ranked relevance measures. If a large proportion of features of * one* property occur close to the top of the list, then * all* the neighbouring pixels over the 3 × 3 grid in that property should be included in the vector.

### 4.4. Training of the SVM

**Figure 6:**
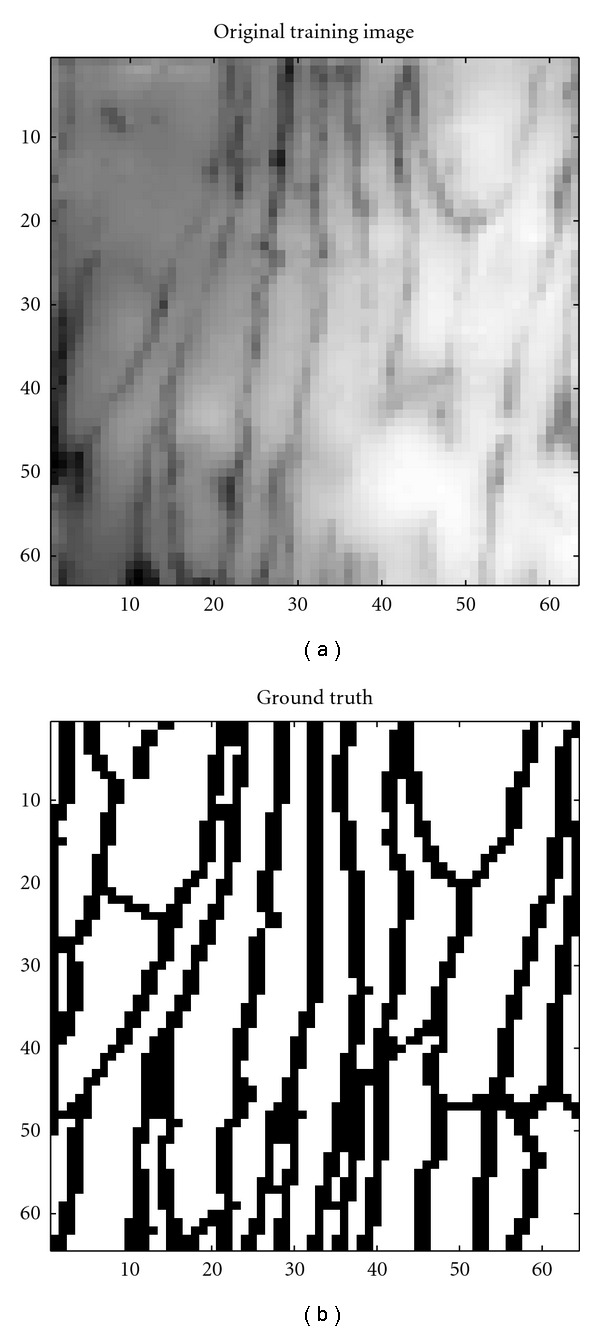
Original training image with its corresponding manually traced ground-truth image.

This algorithm is trained with a sample image of size 62 × 62 pixels taken from one of the 56 regions, as illustrated in Figure 6. The 33-dimensional feature vectors generated over this region are used as an input to the SVM that classifies image pixels into boundary and non-boundary pixels. A binary image is generated after classification with each black pixel corresponding to a boundary and each white pixel corresponding to a non-boundary pixel [13, 18].

For training, we used the RBF kernel [19, 20] with the maximum cost function/parameter set at 1 and *γ* = 0.14. With these settings, 2144 support vectors are produced. The feature extraction process for the training phase took approximately 130 seconds. The algorithm was written with the help of the LIBSVM package [20] in Matlab R2009b [21], and it took 13.38 seconds on a desktop PC with Pentium(R)4 2.8 GHz processor running Windows. The time taken to classify an image of size 512 × 512 is approximately 12–17 seconds, if the features have already been extracted.

## 5. Results

**Figure 7:**
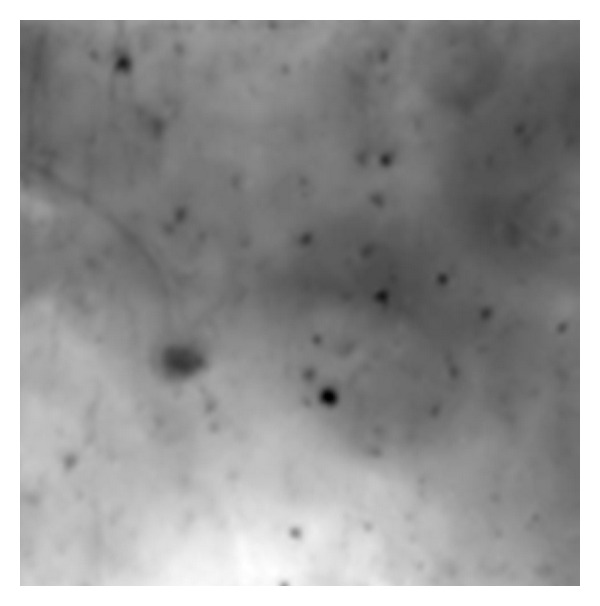
An example of a low contrast and blurred image on which the SVM technique did not work well; note that cell boundaries are not easily identifiable by eye.

**Figure 8:**
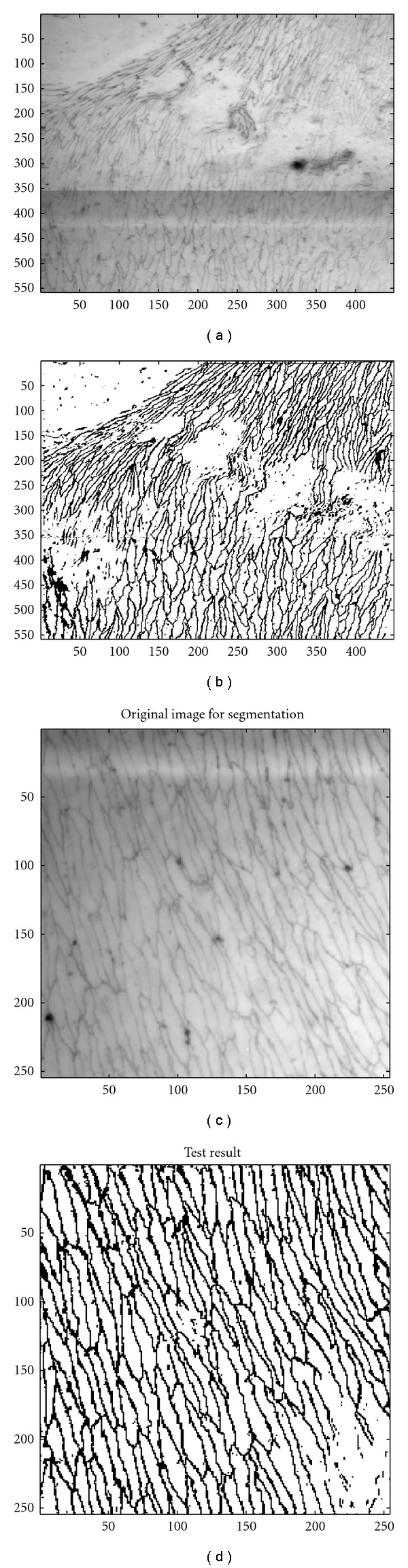
Original image and segmented result using RBF kernel. No other processing has been applied to the SVM output.

The algorithm provided usable boundary data in 43 out of the 56 regions. In the remaining 13 regions, image data are very noisy. One such image is shown in Figure 7, and it may be noted that even discerning the boundaries by eye is difficult; high-pass filtering such an image does not improve matters. The binary images in Figures 8(b) and 8(d) show classifications of pixels into boundaries and background by the SVM algorithm.

### 5.1. Evaluation of the Segmentation Algorithm

The performance of the SVM approach in segmenting cell boundaries was evaluated on four images containing manually traced boundaries; one of these images is shown in Figure 1(b). The manually traced boundaries provide * ground-truth* data that allows the accuracy of the segmentation to be evaluated on a pixel-by-pixel basis. Four trained SVM kernels were applied to each of these test images, and the results were used to determine performance. In total, the performance of the algorithm was assessed on 3.9 million pixels. The parameter, *γ*, was varied from 0.02 to 0.14 across all four SVM kernels in step sizes of 0.01. The RBF kernel reached its best accuracy of 93% at *γ* = 0.14 and the worst result it achieved on a single image was 80%. A comparison between different kernels is given in Table 3. Accuracy of segmentation was assessed by noting the number of correctly classified pixels (either boundary or background) and is defined by

(3) $$\begin{array}{c} \begin{array}{c} \text{Accuracy}\%=\frac{\left(\text{TP}+\text{TN}\right)}{\left(\text{TP}+\text{TN}+\text{FP}+\text{FN}\right)}, \end{array} \end{array}$$

where TP, FP, TN, and FN are true positive, false positive, true negative, and false negative, respectively. 

**Figure 9:**
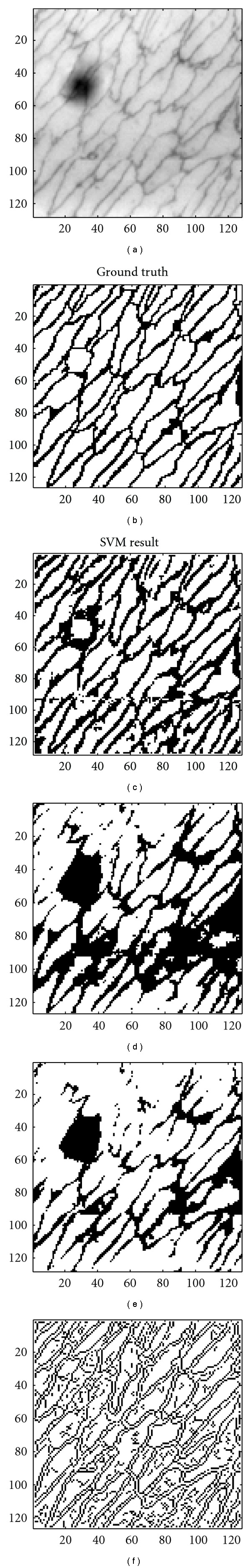
Comparison of results from different image segmentation techniques.(a, b, c): original image with its ground truth and SVM segmentation results. (d, e, f): Kittler method, Otsu method, and Canny edge detection results.

**Figure 10:**
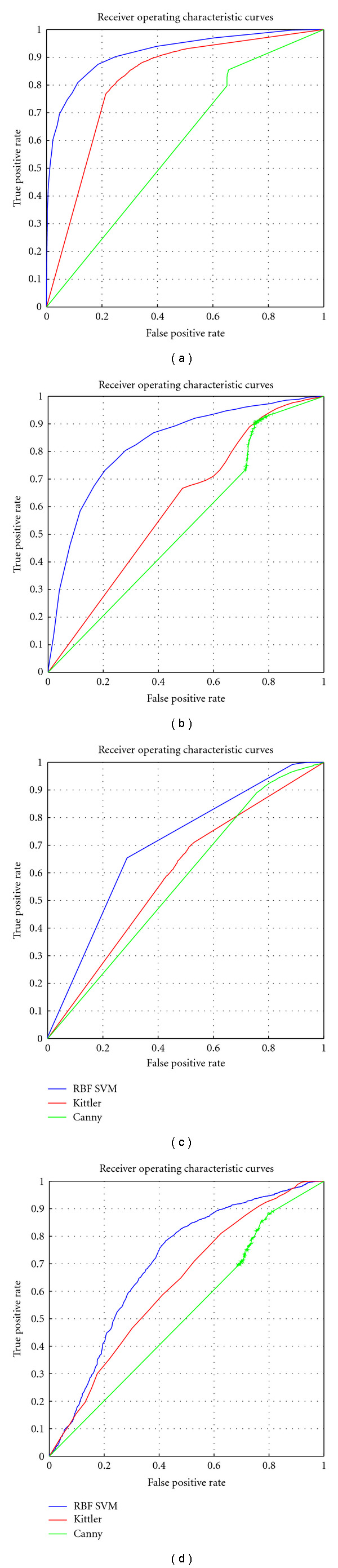
ROC plots for standard Canny and Kittler methods with the SVM (RBF kernel) on four sample images. The performance of the SVM is better, even when applied to the extremely difficult images of the bottom panels.

In another experiment, we compared “ground truth”, an SVM with RBF kernel and two conventional segmentation algorithms, Canny [22] and Kittler [7], in four image patches of size 128 × 128 pixels. The Kittler method was ranked as the best in a survey on thresholding techniques [23]. Different thresholds were set for the decision in order to obtain a number of points along a Receiver Operating Characteristics (ROC) curve, which describes the performance of a classification method and feature space in addressing a discrimination task as one alters the balance between type I and type II errors. For the Canny and Kittler algorithms, 961 and 63 possible detection thresholds were applied to each image, respectively. The SVM model accuracy is largely based on the selection of the model parameters so, to find points along a ROC, a search algorithm was used to attempt training and classification as values of the most significant parameters are altered across a wide range. For example, as the RBF kernel depends on two main parameters (*C* and *γ*), a range of −4 to +4 with interval of 0.1 for *C* and from 0.1 to 2 with an interval of 0.001 for *γ* was selected. However, since each image to be assessed might have different statistical characteristics, generating an ROC might require slightly different parameter settings. For a given image, the model was further evaluated with 5-fold cross-validation to find the best possible combination of parameters.  Table 4 and Figure 9 present the classical segmentation technique results in comparison to the SVM with RBF kernel, and the ROC plots are given in Figure 10.  Table 4 also includes a comparison with the Otsu method [24], which employs an automatic thresholding and is therefore not easily amenable to ROC analysis.

## 6. Discussion

This paper presents a method for detecting EC boundaries that reduces the need for human intervention. Previously, cell boundaries have been segmented manually [9, 25], since noise and unclear or faint boundaries would have led to the failure of traditional automated segmentation techniques. Manual analysis is time consuming, subjective, and unscalable. Previous reports have described computational work on corneal endothelial cell boundary detection [26–29], but the detection of cell boundaries was not as challenging as the EC boundaries in our images. In our previous work [12], we suggested some approaches to automatic cell boundary detection. To address issues of accurately finding edge maps with this previous method, we developed the current method, which has successfully worked on image data with a poor signal-to-noise ratio (SNR). Our algorithm uses small neighbourhood patches in intensity, and orientation dominance patches of each candidate pixel in the image, and successfully segmented 43 out of 56 available regions to an acceptable level of accuracy. An adequate number of cells was characterised for further automatic morphological analysis.

Our method has some limitations: it does not perform well on image patches where the brightness variation is very large or where cell boundaries are not visible by eye. We have found that illumination correction, such as by high-pass filtering, does not improve results and may even worsen them slightly if pixel noise is amplified. The method requires good manual labelling of one example as input in the training process. This necessitates selecting a patch with a representative variation in intensity to yield a satisfactory generalised model. Another aspect of our algorithm which could be improved is the selection of kernels. We have only worked on kernels already available in LIBSVM, but performance might be improved by developing a new kernel for such data [30], which was beyond the scope this research. 

Tests in other types of biomedical image data, such as retinal images, have suggested that with a comparable training process and comparable features, similar promising segmentation results can be achieved with minimal effort. Future work will seek to extend the segmentation method to other biomedical image problems. The method we developed will enable us to explore the relationships between blood flow and cell behavior, between cell nuclear shape and cell boundary shape, and between length : width ratio and cell orientation in different regions around arterial branches, by allowing large numbers of cells to be analysed.

## Figures and Tables

**Table 1:** Six statistical features generated from the 3 × 3 intensity neighborhood of a candidate pixel.

Feature	Formula
Median	$\text{Med}(x,y)=\text{med}\left({\text{I}}_{(i)\sqrt{n}\times \sqrt{n}}(x,y)\right)$

Range	Range(*x*, *y*) = (*Max* (*x*, *y*) − *Min* (*x*, *y*))
	$\text{where Max}(x,y)\;=\max\;\left(I_{(i)\sqrt{n}\times \sqrt{n}}\left(x,y\right)\right)$
	$\mathrm{Min}\;(x,y)\;=\min\;\left(I_{(i)\sqrt{n}\times \sqrt{n}}\left(x,y\right)\right)$

Energy	$E(x,y)=\sum_{i=1}^{n}I_{(i)\sqrt{n}\times \sqrt{n}}^{2}(x,y)$

Second, third and fourth order moments	$M_{r}=1/n\sum_{i=1}^{n}{\left(I_{(i)\sqrt{n}\times \sqrt{n}}(x,y)-\mu (x,y)\right)}^{r}$
	$\mu (x,y)=1/n\sum_{i=1}^{n}I_{(i)\sqrt{n}\times \sqrt{n}}^{}(x,y)$ ,
	*I*(*x*, *y*) is the image, *r* = 2,3, 4: second, third and fourth order moments, *n* is the number of nearest neighbors (in our case this is 9), and *i* is the candidate pixel

**Table 2:** Comparison of minimum redundancy and maximum relevance in the feature vectors between different training data sets is given in Figure 6. We used four different SVM kernels, but the relative relevance attributed to different features is comparable with all kernels.

	Minimum redundancy, maximum relevance feature order in feature vector from highest to lowest
Training dataset	[27 33 3 16 19 32 29 31 21 25 12 31 7 18 26 10 20 1 30 24 9 22 17 11 6 23 15 2 13 8 4 14 5 28]

**Table 3:** Comparison between SVM kernels with varying *γ* in steps of 0.01 in each model. The table is obtained from four different images representing a total of approximately 4 × 10^6^ pixels and four different SVM Kernels. The number of training data pixels was 3844.

*γ*	Accuracy%			
	RBF	Sigmoid	Polynomial	Linear
0.14	92.97	90.06	63.49	55.67
0.13	92.52	88.26	64.53	55.67
0.12	92.82	85.46	65.92	55.67
0.11	92.92	82.55	67.68	55.67
0.10	92.96	79.34	69.63	55.67
0.09	88.95	76.47	71.63	55.67
0.08	91.00	74.19	73.85	55.67
0.07	90.49	71.64	76.43	55.67
0.06	88.14	63.77	79.49	55.67
0.05	88.85	69.70	83.50	55.67
0.04	88.67	64.03	88.61	55.67
0.03	80.66	62.59	83.35	55.67
0.02	82.75	61.67	73.19	55.67

**Table 4:** Comparison between SVM and different conventional methods. Here the number of training pixels was 3844 with 2 classes, and the number of test pixels was 16384.

Method	Accuracy%
SVM with RBF kernel	94.09
Otsu [24]	82.22
Kittler [7]	82.21
Canny [22]	71.83
